# Ultrasonic techniques to obtain dental pulp from impacted third molars

**DOI:** 10.4317/jced.56658

**Published:** 2021-01-01

**Authors:** Jorge G. Becchio, Roque O. Rosende, Jorge E. Monzón, Darío Fernández, Patricia B. Said-Rücker

**Affiliations:** 1 Cirugía III Traumatología Bucomaxilofacial, Facultad de Odontología, UNNE, Argentina. (Surgery III Oral-Maxillofacial Traumatology, UNNE, Argentina); 2 Cirugía I Técnicas Quirúrgicas y Anestésicas, Cirugía III Traumatología Bucomaxilofacial, UNNE. (Surgery I-Surgical and Anesthetic Techniques, Surgery III Oral-Maxillofacial Traumatology, UNNE, Argentina); 3 Grupo de Ingeniería Biomédica UNNE, Argentina (Biomedical Engineering Group, UNNE, Argentina); 4 Laboratorio de Biología Molecular, UNNE, Argentina. (Molecular Biology Laboratory, UNNE, Argentina); 5 Consejo Nacional de investigaciones Científicas y Técnicas. CONICET (National Scientific and Technical Research Council, Argentina)

## Abstract

**Background:**

In the dental clinic impacted teeth are frequent findings, especially upper and lower third molars, leading to their exodontia. Among surgical techniques piezosurgery is advantageous for delicate structures in the oral cavity. Extracted teeth, usually discarded, have been revalued as biological material, providing living tissues with possible applications in regenerative dentistry. The aim was to compare cross-section methods of upper included third molars by ultrasonic piezoelectric technique to obtain dental pulp, with diamond-coated tip (DT) against titanium nitride-coated tip (TN), according to the pulp tissue cell viability and the section surface characteristics.

**Material and Methods:**

Patients attending dental consultation were evaluated. Upper third molars (n= 24) were avulsed from 15 patients with exodontia indication, age 18-26 years old, who agreed to participate of the study. Third molars were cross-sectioned at amelocemental junction level with piezoelectric device using DT or TN inserts. Pulps were mechanic and enzymatically treated, and tissue viability determined by Trypan Blue test. Sectioned teeth were visualized using Scanning Electron Microscope (SEM). Ethical principles of biomedical research were respected; all patients gave their informed consent.

**Results:**

Viability of pulp tissue was 84.71% not associated to sex (p= 0.141) nor to teeth position, upper right third molar or upper left third molar (p= 0.580). According to the insert used, pulp tissue viability was 85.21% with TN, similar to 84.00% with DT (p= 0.611). By SEM, cut performed by TN insert showed smooth and uniform surfaces, while DT insert surfaces were irregular, porous, with fissures.

**Conclusions:**

Piezosurgery applied to cross-section upper third molars with both types of inserts showed differences in the cut surfaces but similar effectiveness regarding preservation of pulp tissue with high viability, thus, they could be allocated for further cellular developments.

** Key words:**Impacted teeth, third molars, piezosurgery, regenerative dentistry.

## Introduction

In the field of dental clinic retained, included or impacted teeth are frequent findings. Impaction refers to a partially or completely unerupted tooth, in an abnormal position against another tooth, bone or soft tissue, so that further eruption to its anatomically position is unlikely (1,2).

Although any tooth of the oral cavity may be affected, third molar impaction occurs frequently in adolescents and young adults; some variation has been reported between females and males, and also among races (1,3).

Since impaction may be more common now than in the past, different theories have been raised in order to explain the appearance of retained third molars based on the masticatory activity, or on evolutionary tendencies of modern humans. Third molar eruption and continuous positional changes afterwards may be related to factors such as nature of the diet, use degree of the masticatory apparatus and to genetic inheritance (3,4). As modern diet tends to be softer, they do not offer a decided effort in mastication, resulting in loss of growth stimulation of jaws, and thus, the modern man has impacted and unerupted teeth (1). It has been suggested that the gradual evolutionary reduction in the size of the human mandible and/or maxilla has resulted in too small mandible or maxilla that may accommodate the corresponding molars, or due to insufficient development of the retromolar space (1,4).

Several pathologies such as caries, pericoronitis, cysts, tumors, also root resorption of adjacent tooth, and inflammation of the opposing soft tissue, have been associated with impacted third molars, causing pain, swelling, and infection, though they may also remain asymptomatic (1,4).

This situation has led to the avulsion of impacted or retained third molars as a frequent procedure in dental practice due to the above mentioned changes in dental topography, along with other causes. Moreover, removal of impacted third molar is one of the most common procedures in oral and maxillofacial surgery. Several classification systems are used to examine the presence and impaction state of these teeth, among them, Winter´s considers the angulation of the third molars, and the Pell and Gregory system takes into account the position of the impacted third molars in relation to the occlusal plane (5).

Exodontia of teeth has evolved, improving surgical techniques such as piezoelectric bone surgery, called piezoelectric or piezo surgery, which is a bone cutting system based on ultrasonic microvibrations. Piezoelectric devices are used widely for osteotomies, such as maxillary sinus lift, impacted mandibular third molar extraction, and bone grafting, in the field of oral and maxillofacial surgery. Moreover, use of piezosurgery for extraction of the impacted mandibular third molar was reported to produce less facial swelling and trismus postoperatively compared to that of rotary osteotomy (6).

Piezosurgery is an advantageous technique for delicate structures in the oral and maxillofacial region, respect to osteotomies of thin and fragile bones the application of ultrasound is superior to other mechanical instruments because of the extremely precise and virtually arbitrary cut geometries, easy handling, efficient bone ablation and minimal accidental damage to adjacent soft tissue structures (7). It is helpful to perform bone cutting with great precision facilitating ridge augmentation and ridge expansion, tooth extraction, ankylotic tooth extraction and surgical orthodontic surgeries, while precluding injury to soft tissue, minimal heat is generated during cutting, thus maintaining vitality of adjacent tissue (8).

*Pi*ezoelectric tips do not require pressure on bone to be effective so thermal injuries and bone microfractures are reduced. The piezosurgery tips can be titanium nitride-coated (TN) or diamond-coated (DT), showing different characteristics (9).

The extracted teeth, which were usually discarded, today have been revalued as biological material since they can provide living tissues with possible applications in regenerative dentistry. Feasible available adult dental pulp stem cells are an excellent resource for cell therapy and can be obtained from orthodontic extraction teeth or wisdom teeth (10).

The aim of this study was to compare cross-section methods of impacted upper third molars by ultrasonic piezoelectric technique, with TN tip against DT tip, to obtain dental pulp according to their resulting tissue cell viability and section surface characteristics.

## Material and Methods

Patients: between September 2016 and December 2018 patients attending dental consultation were evaluated at the Faculty of Dentistry, Northeast National University (UNNE), in Corrientes city, Argentina. For each case the clinical record was generated as the patient was clinically assessed, including observations related to oral health. The clinical and imaging evaluation of the patient included panoramic radiographic images, computed axial tomography (CAT scan), dental scan, and cone beam system, also pre-surgical laboratory study, with clotting and blood analysis. Among all the patients evaluated within the period, in 23 of them exodontia was indicated, with average age of 23.35 (from 16 to 52) years old, being 60.9% of them women. Only two patients were married, while the rest were single. Regarding the occupation, there was a housewife, a lawyer and 91.3% were students. A total of 43 third molars were avulsed from the patients.

Impacted third molars: teeth were included in the study if they were extracted from patients with exodontia indication, adequate oral hygiene, in absence of supra or infra-gingival stones, within an age range of 18 to 26 years old, who agreed to participate of the study by donation of the avulsed teeth, giving their informed consent (including criteria).

Sample of teeth: consisted of 24 upper impacted third molars avulsed from 15 patients with exodontia indication.

Avulsion of impacted third molars: were performed using anesthesia of Carticain Hydrochloride 4% L-Adrenaline 1:100,000 by vestibular puncture at the upper anterior alveolar nerve level and by palatine to the anterior palatine nerve, followed by incision with scalpel number 15c, from distal of second molar, inclined towards the palatine on the bone crest to the terigomaxillary mucosal fold, with a small discharge by mesial or distal from 7 (second upper molar).

Flap lifting: is mucoperiostic with movement from front to back, the osteotomy was performed with a gouge or periostotome forceps if bone was very thin, when retentive bone was thicker cylindrical or round surgical burrs nº6 were used, the tooth was dislocated with straight or angled elevators, manipulating with forceps number 67. For treatment of the socket a bent curette was used, dental follicle residues (pericoronary sac) were removed with a curved mosquito clamp (Halsted), cavity bony edges were smoothed down with bone files, and final wash of the cavity was done with profuse irrigation using 0.9% saline solution and 0.12% chlorhexidine gel. After repositioning the flap, simple stitches were performed with Nylon 4/0.

Patient follow-up: After avulsion, post-operative indications were informed in writing and explained to the patients. Stitches were removed after 7 days.

Conservation of removed teeth: they were immediately placed in cold Dulbecco´s Modified Eagle Medium –DMEM (Gibco) to ensure cellular preservation of the pulp tissue.

Cross-section methods: by ultrasonic techniques using NSK piezoelectric device (Vario Surg Ultrasonicm) within a biological safety cabinet, set at piezosurgery position, option C, with 100% torque and 50% of its potential irrigation. Once TN or DT insert was placed, each tooth was hold with bracket forceps and cross-section was performed at amelocemental junction level. It started by marking all the circumference of the amelocemental boundary with forth and backward movements to allow a more precise cut to 2 mm depth. Then pressure was applied at the mark to the teeth, hold with orthodontic pliers within the palm of the hand, and it was fractured at the cervical level into coronary and radicular parts.

Pulp tissue extraction: once the tooth was cut, the pulp was gently detached with Black’s spoon from the pulp chamber and root canals. Then, the dental pulp was removed with the excavator and placed in an Eppendorf tube with 500 μl of DMEM solution at 4° C. Dental pulps were mechanically sectioned using 15c scalpel in Petri dish and fragments were placed in Eppendorf tubes for enzymatic digestion with 300 μl Collagenase/Dispase (Roche) solution for 1 hour at 37 ° C, the process was stopped on ice.

Cell viability test: Trypan Blue is a dye used to evaluate the cell viability; living cells with intact membranes exclude the dye, whereas dead cells do not (11). Cell count was performed by adding 0.4% trypan blue solution to cell suspension, after 2 minutes the mixture was placed in each grid of the hemocytometer or Neubauer chamber. Blue cells (dead) and birefringent or white cells (live) were counted separately under light microscope. Proportion of living cells related to total number was reported.

Scanning Electron Microscope (SEM): cross-sectioned third molars were prepared for viewing by metallization in the Sputtering Denton Vacuum Desk II. Visualization was carried out with JEOL 5800LV Scanning Electron Microscope, analysis was performed using the Gatam MODEL 788 Digiscan II Digitizer at the UNNE Scanning Electron Microscopy Service.

Statistical analysis: data in electronic files was analyzed using statistical package SPSS 15.0 for Windows. Student test and variance analysis were used to determine differences between normally distributed means.

Ethical considerations: the study was performed under the approval reference Number 101/2016 of the Bioethics Committee, and the Resolution Number 231/16 of the Directive Council, both belonging to the Faculty of Dentistry, UNNE. Ethical principles of biomedical research were respected; each patient included in the study was fully able to grant free and voluntary consent for their participation. Privacy was guaranteed to the material (teeth), information and personal data that they provided according to legislation in force in Argentina.

## Results

Patients attended dental consultation referring pain close to the third molar level, at the temporomandibular joint; symptoms appeared at one or both sides of the head. Clinical findings were related to impacted third molars, including tissue inflammation, dental crowding (undergoing or not orthodontic treatment), molar pericoronitis that hindered mastication and impairment of dental occlusion, among others. Thus, due to different reasons exodontia procedure was indicated to the patients.

Exodontias were performed in operating room to 23 patients, and a total of 43 third molars were avulsed, resulting in 17 upper right third molars, 16 upper left third molars, 4 lower left third molars and 6 lower right third molars. According to inclusion criteria, lower third molars were excluded, also other 3 since they were cut by other device, as well as 6 more, destined to culture in which viability was not assessed. Finally, 24 upper right third molars or upper left third molars were included (12 of each), extracted from 15 patients.

**Figure 1:**
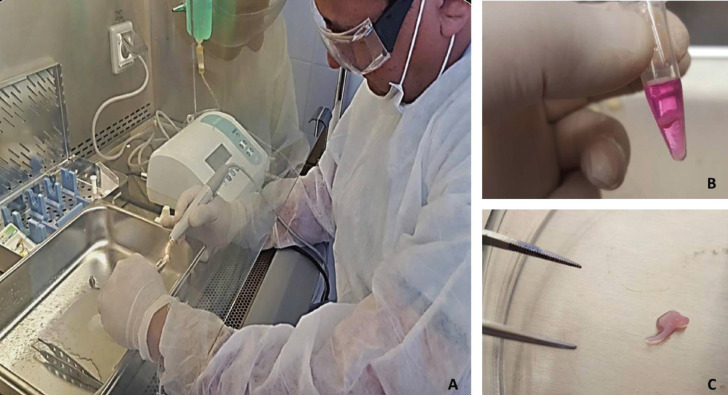
Piezoelectric cross-section of a tooth within a biological safety cabinet (A); extracted dental pulp in DMEM (B); dental pulp in Petri dish (C).

Regarding the cross-section of the teeth with the piezoelectric device, TN-coated tip was used in 58.3% of the cases (n = 14) and DT-coated tip in the other ten (Fig. 1A). Having used inserts with the same intensity of torque and irrigation, it was observed that the cross-section of the teeth with DT tip was slower unlike with the TN tip. Once third molars were cut, the dental pulps were removed and processed by mechanic section and enzyme digestion, prior viability test as seen in Figure 1 B to C.

Dental pulps showed an average viability of 84.71% (± 5.36), in a range of 73% to 93%. Viability of pulp tissue was not associated to the sex of the donors as seen in Table 1.

**Table 1:**
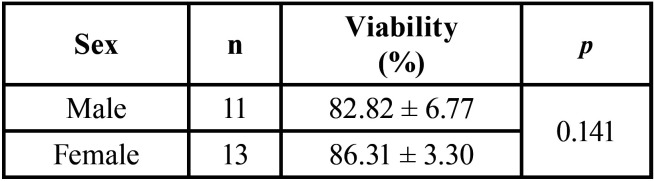
Dental pulp viability stratified by sex.

Pulp cellular viability was studied according to the oral cavity position of the tooth, upper right third molar or upper left third molar. The difference was not significant as seen in Table 2.

**Table 2:**
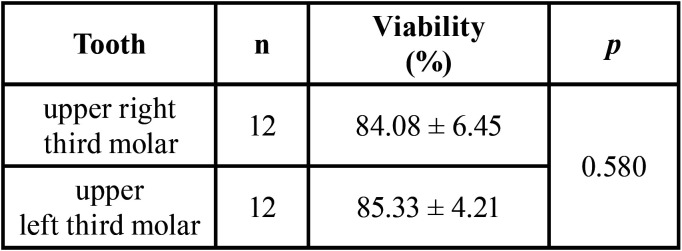
Pulp viability stratified by tooth position.

It was studied if the cell viability of the pulps was related to the insert used to make the cross-section of the tooth. However, there was no association (Table 3).

**Table 3:**
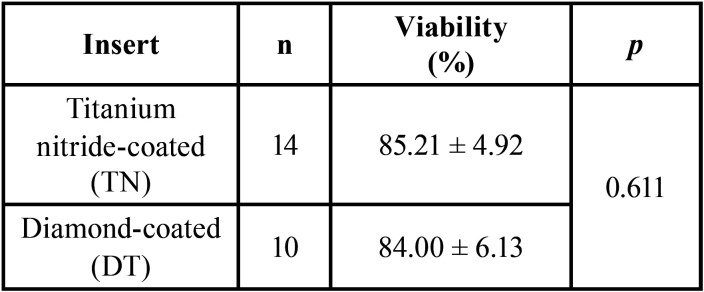
Pulp viability stratified by tooth position.

Surfaces of cross-sectioned third molars were visualized by SEM images. Scales in micrometers (µm) were included to the images for size reference.

**Figure 2:**
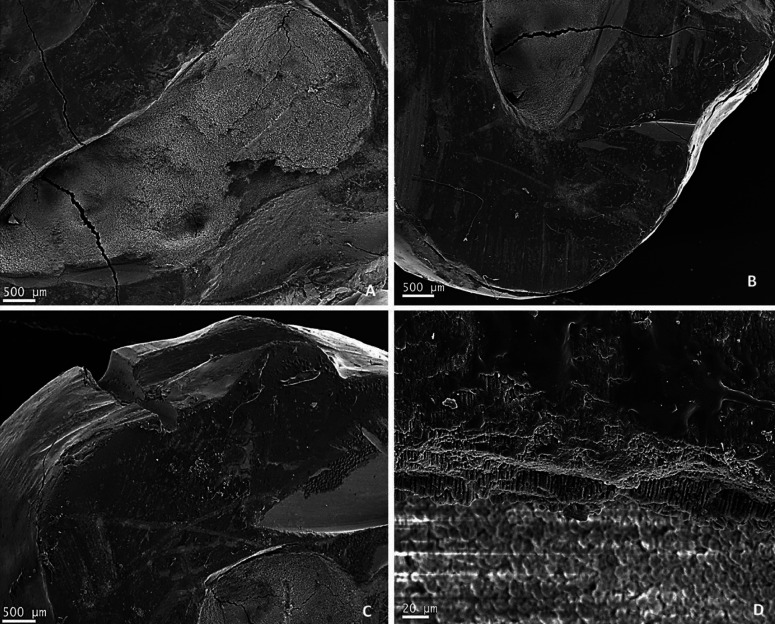
JAF patient upper left third molar: pulp chamber view (A); cut surface around the pulp chamber (B and C); detail view of amelo dentinal limit, dentinal canaliculi and the entrances of mesio vestibular and disto vestibular ducts (D).

Upper left third molar belonging to JAF patient was sectioned with NT piezoelectric tip. Figure 2 shows different areas of the cut surface around the hollowed pulp chamber, and details of amelodentinal limit. By SEM the third molar displayed smooth and uniform surfaces.

**Figure 3:**
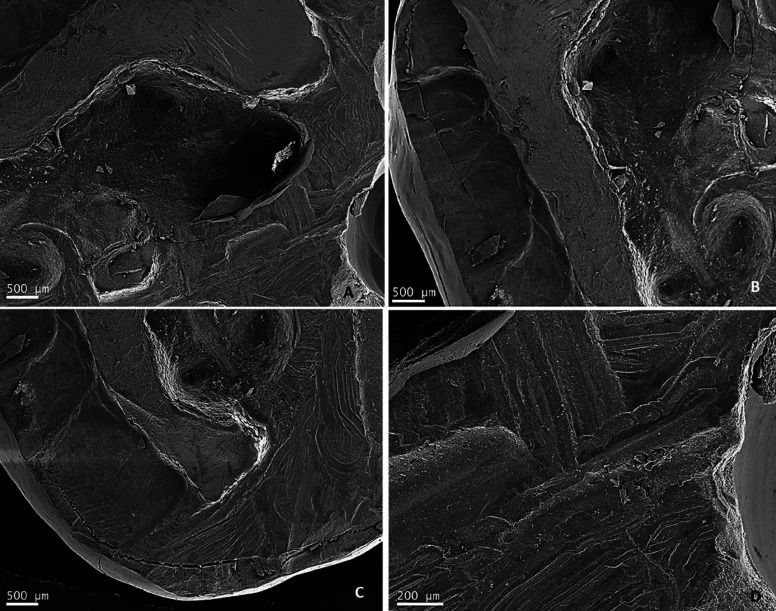
SV patient upper left third molar: pulp chamber view (E); cut surface around the pulp chamber (B and C); closer view of cross-section surface (D).

SV patient upper left third molar was cross-sectioned with DT piezoelectric tip. In Figure 3 the pulp chamber, surrounding cut surfaces, dentinal canaliculi entrances and details of the surface are shown. This third molar displayed irregular and porous surfaces, with small fissures and fracture lines by SEM.

## Discussion

Patients who constituted the clinical cases of the present study attended dental consultation due to pain at the end of the dental arch or at the temporomandibular joint, others showed crowding, and were referred to practice avulsion of the third molars for orthodontic treatment. Partially erupted or total retained third molars can generate disorders that require immediate dental attention to find the best treatment options, so exodontia was indicated based on clinical evaluation and complementary studies. These findings were consistent with other studies that refer pain as a symptom that evidences a problem that can compromise patient’s health and requires dental care (4).

Clinical cases with indication of exodontia were mainly young patients, as described by other authors, with no difference in their age according to sex (1,3).

After removal of the teeth, the post-extraction procedures for pulp retrieval have been done by usual methods to cross-section them such as diamond disc, along with coolant to avoid damage to the tissue (12), or dental fissure burs to decoronate the tooth and expose the pulp chamber by cutting around the cementoenamel junction, with ample amount of sterile water sprayed to reduce heat (13,14).

Within the framework of this experimental study, working with the impacted upper third molars avulsed from the patients, an innovative technique using ultrasonic vibrations as piezosurgery allowed the extraction of the dental pulps with preservation purposes for new developments. Tooth cross-section at amelocemental junction level was the most appropriate option since it was adapted to morphology curvatures found in dental practice (7).

Piezosurgery method compared to rotary bur for third molar removal has been found to reduce postoperative pain, trismus, and swelling, as well as, it may play a relevant role increasing bone density within the extraction socket and decreasing the amount of bone loss of adjacent tooth in clinical practice. As compared to conventional rotary instrument, pain score and swelling score after surgical removal of mandibular third molars were significantly lower with piezosurgery, also mouth opening was better, hence although more operation time was required, there might be some advantages in the last mentioned surgical approach (14).

Dental pulps from third molars showed high viability, between 73% and 93%, the average being 84.71%. There were no differences in the average pulp viability neither according to the donor sex, nor to the location of the tooth in the upper jaw, right or left third molars. Regarding pulp tissue, Naz *et al.* studied the cellular viability of dental pulp stem cells isolated from permanent and deciduous teeth using trypan blue dye exclusion test and reported more than 90% viability in both cases (13).

Impacted upper third molars sectioned by piezoelectric device with the titanium nitride-coated tip showed similar pulp viability with respect to those cut with diamond-coated insert. Pulp tissue preserved high cell viability with both cross-section techniques, indicating that the choice of either insert is valid to obtain pulps suiTable for *in vitro* cultures or other destinations. These results would indicate that piezoelectric is indeed an excellent alternative for delicate structures in the oral cavity, causing little tissue damage as described by authors elsewhere (8,9).

The ultrasonic technique using each type of insert (with equal torque and irrigation conditions) and the third molar cross-section surfaces visualized by SEM, were compared. The procedure lasted more with diamond-coated tip, leaving porous and irregular cutting surfaces, with cracks and fracture lines. On the other hand, section of teeth using titanium nitride-coated tip was accomplished in less time, showing smooth and uniform cutting surfaces. In accordance with these results, Hennet found that diamond-coated tips may be considered less aggressive and safer on thin bone, adequate for gentle bone removal, and therefore the section of hard structures as third molars would take longer (9).

Third molars are pieces of interest for dentistry from multiple perspectives, anatomical, as well as surgical and forensic, among others. The study contributed to realize differences among patients, given that some showed alterations such as crowding or poor occlusion of the third molars. Abnormalities arise from alterations in the odontogenesis process, which may affect the primary dentition, the secondary dentition or both, requiring timely attention to minimize the impact on the patient’s oral health (2).

Due to their high probability of retention, third molars require differentiated dental treatment that often leads to surgery, implying possible complications before and after the procedure. A retrospective study to determine the eruption and impaction state of third molars, with near 3800 patients, showed that vertically impacted maxillary third molars and horizontally impacted mandibular third molars were most frequent in all age groups, in this sense, the authors highlighted that regular oral examination as essential to keep asymptomatic third molars in good health (5).

These teeth worked as an *in vitro* experimental model to study the cross-section of their enamel and dentine by ultrasound using piezoelectric device and its effect on the pulp tissue. Similarly, other experimental studies also used extracted human teeth to describe different types of dental wear through analysis of the macro- and micromorphological features, and to evaluate the different concentrations of sodium hypochlorite activated with laser in removing of the smear layer under controlled conditions, both by scanning electron microscopy (16,17).

Piezosurgery with both types of inserts applied to cross-section upper third molars extracted from young patients, showed differences in the cut surfaces but similar effectiveness regarding preservation of pulp tissue, as evidenced by high cellular viability, thus it could be allocated for further developments in regenerative dentistry.
